# Is there a correlation between functional recovery of manual dexterity after motor cortex lesion and initial motor learning slope in the intact state?

**DOI:** 10.3389/fnsys.2026.1754760

**Published:** 2026-03-17

**Authors:** Eric M. Rouiller

**Affiliations:** Section of Medicine, Department of Neurosciences and Movement Sciences, Faculty of Sciences and Medicine, University of Fribourg, Fribourg, Switzerland

**Keywords:** hand function, learning curve slope, monkeys, motor cortex lesion, motor functional recovery, motor learning, non-human primate

## Abstract

A cohort of 13 adult macaques offered a unique opportunity to collect over several years manual dexterity data, from an initial learning phase in intact animals to a terminal phase of functional recovery after unilateral lesion of primary motor cortex (M1). Manual dexterity was assessed daily using the modified Brinkman Board task, yielding a total score given by the number of food pellets retrieved by one or the other hand from vertical and horizontal slots. A motor *learning curve slope* was established during the initial learning phase before reaching a stable performance with the dominant hand. Later, following contralateral M1 lesion, the manual dexterity score dropped to zero, before a progressive spontaneous functional recovery occurred, reaching a unique plateau of usually incomplete recovery. A *recovery curve slope* was calculated. In six of the 13 monkeys, a treatment aimed at enhancing the functional recovery of manual dexterity was applied, yielding a second plateau of recovery added to the first spontaneous recovery plateau. A *recovery curve slope* was also calculated for the second plateau. The hypothesis that steep initial motor learning is correlated with rapid and efficient functional recovery after M1 lesion was tested. In contradiction to this hypothesis, the data showed an inverse correlation with decreasing *recovery curve slopes* as a function of increasing *learning curve slopes*. This result suggests that the mechanisms underlying initial motor learning may be different from those mobilized for functional recovery after M1 lesion.

## Introduction

**FIGURE 1:**
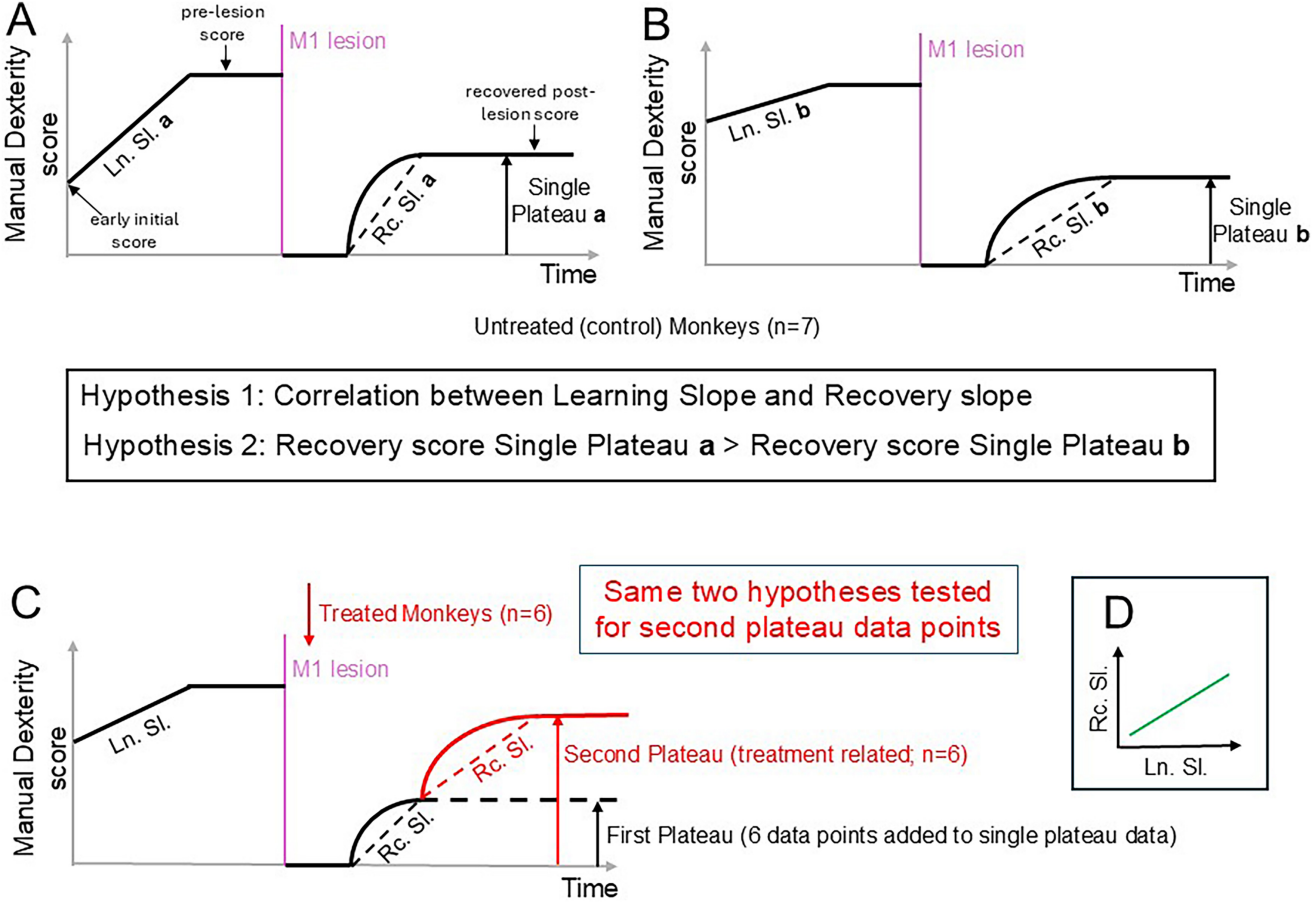
Two hypotheses to be tested. **(A)** Cartoon illustrating the typical time course of manual dexterity score for an adult “untreated” macaque monkey (“monkey a”), based on the modified Brinkman Board task executed with the dominant hand, including the initial motor learning phase, with a steep *learning curve slope* (“Ln. Sl. a”). The end of the learning phase is characterized by a plateau. Then, a unilateral experimental M1 lesion contralateral to the tested hand took place (purple vertical line), which provoked a total loss of manual dexterity during a few weeks. It was followed by a spontaneous, progressive functional recovery, until reaching a single plateau of recovery. The dashed line represents the corresponding *recovery curve slope* (“Rc. Sl. a”). In absence of treatment, the plateau remained stable for months to years. The vertical arrow represents the recovered post-lesion score. **(B)** Same as in panel **(A)**, but for a *gentle learning curve slope* represented by an untreated “monkey b.” Note that both slopes (*learning curve slope* and *recovery curve slope*) are clearly less steep than for “monkey a.” Seven monkeys included in the present study corresponded to such a time course, as depicted in panels **(A,B)**, with various *learning* and *recovery* curve slopes (Table 1). The hypothesis 1 tested in the present report argues that the initial motor learning slope is correlated with the slope of functional recovery from a M1 lesion, possibly following the relationship between *learning curve slope* and *recovery curve slope* as depicted in panel **(D)**. As a consequence, the hypothesis 2 argues that in case of “steep initial motor learning” a higher recovered score of manual dexterity after M1 lesion will be reached than in case of “gentle initial motor learning.” **(C)** Same as in panels **(A,B)**, but for a M1 lesioned monkey subjected to a treatment (either anti-Nogo-A antibody or ANCE autologous cellular therapy; *n* = 6 monkeys). The M1 lesion is indicated by the vertical purple line, followed by the treatment onset (red arrow pointing down). Following the total loss of manual dexterity, a first plateau of functional recovery took place, reflecting an initial spontaneous recovery. This first plateau of recovery was used to derive six additional data points to the seven data points illustrated in panels **(A,B)**. Overall, 13 data points were representative of the spontaneous recovery post-M1 lesion [Table 1; see also (Rouiller, 2026) for more detail]. As a result of treatment, these six monkeys exhibited a second plateau of functional recovery, representing the effect of the treatment (red curve). As for the first plateau, a *recovery curve slope* (“Rc. Sl.”) and a recovery score (red arrow pointing up) can be derived specifically for the second plateau (Table 1), corresponding overall to six “treatment” data points. **(D)** Expected correlation between the *recovery curve slope* and the initial *learning curve slope*, for both plateau 1 and plateau 2 (see above).

As a result of practice or experience, motor learning of a specific, new motor task corresponds to a relatively permanent improvement in performance, until reaching a plateau of stable motor capacity. In a previous report (Kaeser et al., 2014), when confronted for the first time to a manual dexterity task, the modified Brinkman Board task, the motor learning properties of adult monkeys were illustrated and quantified. Young adult intact monkeys were trained to retrieve small food pellets from vertical and horizontal slots, using one or the other hand. As established for their dominant hand (Chatagny et al., 2013; Kaeser et al., 2014), the slope of the motor learning curve was obtained by dividing the gain of performance (number of pellets retrieved) by the time (number of days) until reaching the plateau of performance (Kaeser et al., 2014). Within an original population of 20 monkeys (Kaeser et al., 2014), there was a large variability of motor *learning curve slopes*, ranging from nearly zero (Mk-MO, no learning phase) to 0.42 (Mk-AT, abrupt learning phase). From this original group of 20 monkeys in which the learning phase for the modified Brinkman Board task was quantified, 13 of them were subjected later in their life to an experimental permanent unilateral lesion of the hand representation in the primary motor cortex (M1), as listed in Table 1. Immediately after the M1 lesion, the manual dexterity was totally suppressed (the score dropped to zero). In seven of those monkeys, in absence of any treatment (“untreated” monkeys), a spontaneous and progressive functional recovery from the M1 lesion took place (Figures 1A, B), until reaching a unique plateau of incomplete recovered performance, corresponding to a quite variable percent of functional recovery (Table 1). In the other six monkeys, the functional recovery was tentatively boosted via one or the other of two pilot therapies (Table 1). In these six “treated” monkeys, there was a first plateau of recovery corresponding to an initial spontaneous recovery, followed by a second plateau reflecting the recovery enhancement effect of the treatment (Figure 1C). The raw data for these 13 monkeys were published previously in detail, including illustrations of the manual dexterity performance with time, before and after the M1 lesion (Gindrat et al., 2025; Gindrat, 2015; Kaeser et al., 2011, 2014; Liu and Rouiller, 1999; Roux et al., 2025; Savidan et al., 2017; Wyss et al., 2013). As shown in Figure 1, a *recovery curve slope* can be calculated for both the first plateau and the second plateau of functional recovery.

**TABLE 1:** List of monkeys with their ID (leftmost column) and the volume of the primary motor cortex lesion (2*^nd^* column).

Monkey ID	Les. vol. (mm^3^)	Treatment	2 nd plateau	% rec. T	% rec. V	% Rec. H	Score T post	Score V post	Score H post	Initial score T	Learning slope (T)	Recovery slope (T)
Mk-BI*^a^*	20.1	None	No	68	97	32	21	16.5	4.5	29.5	0.0336	0.6571
Mk-CE*^a^*	112.8	None	No	38	64	9	10	9	1	22	0.274	0.0145
MK-DG*^b^*	32.2	None	No	60	71	43	19	12	6	20.5	0.119	0.5
Mk-DI*^c^*	68.5	None	No	39	79	7	11.5	11	1	20.5	0.410	0.2097
Mk-GE*^a^*	48.7	None	No	42	57	11	10	8	1	18	0.0516	2
Mk-RO*^a^*	14.0	None	No	75	87	80	21	13	10	23.5	0.0288	0.4643
Mk-AN*^d^*	27.7	None	No	65	70	55	13	7	6	9	0.0795	0.5217
Mk-VA - 1st pl.	20.0	Control-like	first plat	50	67	32	13	10	3	24	0.0937	0.941
Mk-MO - 1st pl.	41.8	Control-like	first plat	56	89	25	19	17	4	33.5	-0.0037	1.6666
Mk-JA - 1st pl.	20.5	Control-like	first plat	63	63	54	17	10	7	23	0.0199	0.9
Mk-JO - 1st pl.	30.0	control-like	first plat	34	56	0	11.5	10	0	24.5	0.121	0.6316
Mk-CA - 1st pl.	22.0	Control-like	first plat	50	76	25	15	13	3.5	12	0.0598	0.2429
Mk-LO - 1st pl.	19.1	Control-like	first plat	65	64	67	17	9	8	12.5	0.0827	0.5313
Mk-VA*^e^*	20.0	Anti-Nogo-A	Yes	87	87	73	22.5	13	8	24	0.0937	0.1886
Mk-MO*^e^*	41.8	Anti-Nogo-A	Yes	76	84	56	26	16	9	33.5	-0.0037	0.57
Mk-JA*^a^*	20.5	ANCE	Yes	98	94	100	26.5	15	13	23	0.0199	0.3333
Mk-JO*^a^*	30.0	ANCE	Yes	59	89	25	20	16	4	24.5	0.121	0.2962
Mk-CA*^d^*	22.0	ANCE	Yes	67	71	61	20	12	8.5	12	0.0598	0.0943
Mk-LO*^d^*	19.1	ANCE	Yes	92	93	92	24	13	11	12.5	0.0827	0.0787

The 3rd column from the left indicates the corresponding therapy or treatment: none is for “untreated” (no treatment) monkeys (*n* = 7), “control-like” is for the first plateau (spontaneous recovery) in the “treated” monkeys (see Figure 1C; *n* = 6); overall, the number of “control” data points is 13 (7+6). Two monkeys (Mk-VA and Mk-MO) were treated with an anti-Nogo-A antibody, whereas four monkeys were subjected to the ANCE cellular therapy: overall, the pooled treated monkeys yielded six “treated” data points corresponding to their second plateau of functional recovery. Note that the learning curve slope (second column from right), before M1 lesion, obviously was the same for the six treated monkeys for their first or second plateau data line. The 4th column from the left indicates the absence (No) or presence (Yes) of a second plateau of functional recovery of manual dexterity; in case of the presence of a second plateau of recovery, the first plateau was considered as spontaneous recovery [see also (Rouiller, 2026)], thus yielding an additional “control-like” (i.e., no treatment) data point for the corresponding monkey (lines with gray characters). Data points in black or gray thus represent spontaneous functional recovery, while data points in red or green represent treatment enhanced functional recovery (anti-Nogo-A antibody or ANCE, respectively). The nine rightmost columns list the nine behavioral parameters considered in the analysis (see text). In these nine columns headings, “T” is for the Total number of slots, “V” is for the number of Vertical slots, and “H” is for number of Horizontal slots. “% Rec.” is for the percentage of functional recovery. The scores post-lesion (“post”) are given by their median values, either at first plateau (*n* = 13) or at second plateau (*n* = 6), separately for the total number of slots (“T”), the vertical slots (“V”), and the horizontal slots (“H”). The “Initial Score T” computed for the total number of slots corresponds to the score estimated for each monkey before starting the motor learning phase in the intact state [derived and rounded based on Kaeser et al. (2014): Table 1]. The learning curve and the recovery curve slopes were computed for the total number of slots (“T”). Original data (pre-lesion plateau and post-lesion plateau) derived from:

*^a^* Kaeser et al., 2011,

*^b^* Savidan et al., 2017,

*^c^* Gindrat, 2015; Gindrat et al., 2025,

*^d^* Roux et al., 2025,

*^e^* Wyss et al., 2013. The learning curve slope data are those originally reported in Kaeser et al. (2014) (see Table 1). The recovery curve slopes were calculated as illustrated in Figure 1 and listed here based on the behavioral data (total number of slots) previously published (^a–e^ above). The recovery curve slopes are the only new data introduced in the present report, but they were computed based on previously published data (^a–e^ above). In our large collection of monkeys, two more monkeys (Mk-LA and Mk-SL) were subjected to M1 lesion and anti-Nogo-A antibody treatment. However, they were not included in the present report because the initial motor learning data were not available. Sex: Mk-DI, Mk-GE, Mk-AN, Mk-CA, Mk-LO were female macaques whereas Mk-BI, Mk-CE, Mk-DG, Mk-RO, Mk-VA, Mk-MO, Mk-JA, Mk-JO were males. Age: Most monkeys’ age at M1 lesion time ranged from 3.5 to 5 years old, except the older monkeys Mk-DI (9.5 y), Mk-DG (9.5 y), Mk-AN (14 y), Mk-CA (11 y) and MK-LO (11.5).

The initial motor *learning curve slopes* and the subsequent functional *recovery curve slopes* from M1 lesion are listed for the 13 monkeys in Table 1. Capitalizing on such a rather unique opportunity to confront, for the very same motor task (modified Brinkman Board task) and in the same individual, the motor *learning curve slope* in the intact state and the functional *recovery curve slope* post-M1 lesion, one can address the following question: is there a relationship between initial learning slope and functional recovery slope post-M1 lesion?

One may be tempted to expect a correlation between the two slopes, with probably “steep initial motor learning” in the intact state associated to a more rapid functional recovery in case of M1 lesion occurring later in life, as illustrated in Figure 1D (hypothesis 1). Furthermore, “steep initial motor learning” may be correlated with a higher plateau of functional recovery after M1 lesion late in life (hypothesis 2 in Figure 1).

## Materials and methods

Manual dexterity was investigated and quantified in adult monkeys (macaca fascicularis) on the basis of our modified Brinkman Board task, as previously reported in several articles from this laboratory (Chatagny et al., 2013; Gindrat et al., 2025; Gindrat, 2015; Hoogewoud et al., 2013; Kaeser et al., 2011, 2014; Liu and Rouiller, 1999; Rouiller et al., 1998; Rouiller, 2026; Roux et al., 2025; Savidan et al., 2017; Schmidlin et al., 2011; Wyss et al., 2013) and derived from previous versions of the task (Brinkman and Kuypers, 1972, 1973; Brinkman, 1984). Briefly, a manual dexterity daily score was calculated, given by the number of pellets successfully retrieved from vertically oriented slots and from horizontally oriented slots during the first 30 s of the test, yielding a “vertical” score and a “horizontal” score; furthermore, a “total” score was derived, given by the sum of the vertical and horizontal scores (Supplementary Video 1). After M1 lesion, following a transient total loss of manual dexterity (as illustrated in Figure 1), a progressive functional recovery was observed, reaching a single plateau in “untreated” monkeys, while “treated” monkeys exhibited two plateaus of recovery. For each plateau, a percentage of functional recovery was calculated, dividing the post-lesion median score by the pre-lesion median score × 100. The variability of the daily behavioral scores established with the modified Brinkman Board task was quantitatively reported in a recent report (Rouiller, 2026). The precise housing conditions of the monkeys in the animal facility were reported in detail earlier (Kaeser et al., 2014); see also www.unifr.ch/spccr/about/housing.

At a relatively young age [range 3–7 years old; weight 2.5–5.2 Kg; see (Kaeser et al., 2014)], the monkeys were first exposed to the modified Brinkman Board task, exhibiting for most of them a motor learning phase, characterized by a progressive increase in performance, until reaching a plateau of stable performance. This learning phase was used to derive for each monkey a *learning curve slope* (number of pellets increase divided by the duration in days of the learning phase), as illustrated earlier (Kaeser et al., 2014) and reminded here in Table 1 for the 13 monkeys included in the present study. Based on their behavioral data previously published (Gindrat et al., 2025; Gindrat, 2015; Kaeser et al., 2011; Liu and Rouiller, 1999; Roux et al., 2025; Savidan et al., 2017; Wyss et al., 2013), for the present study an original *recovery curve slope* was calculated as illustrated in Figure 1, for both the unique/first plateau and the second plateau of functional recovery (listed in Table 1 for each monkey).

In the 13 monkeys subjected to a unilateral M1 lesion, seven exhibited a spontaneous functional recovery, as they were not subjected to any treatment (“untreated” monkeys; see Table 1). In contrast, six monkeys were “treated,” either with an anti-Nogo-A antibody (Freund et al., 2006, 2007, 2009; Hamadjida et al., 2012; Hoogewoud et al., 2013; Schwab, 2004, 2010; Wyss et al., 2013) or with the ANCE autologous cellular therapy (Bloch et al., 2014; Borgognon et al., 2017, 2019; Brunet et al., 2005, 2009; Kaeser et al., 2011; Roux et al., 2025). The M1 permanent lesions, induced chemically by infusion of ibotenic acid, were histologically reconstructed, represented on lateral views of the brain (Gindrat, 2015; Kaeser et al., 2011; Liu and Rouiller, 1999; Roux et al., 2025; Savidan et al., 2017; Wyss et al., 2013) and, finally, a lesion volume was calculated for each monkey (Table 1). All these methods (M1 lesion procedure, lesion reconstruction and treatments) were described in great detail earlier (see articles from this laboratory mentioned above) and are therefore not repeated here in the current brief research report.

## Results

**FIGURE 2:**
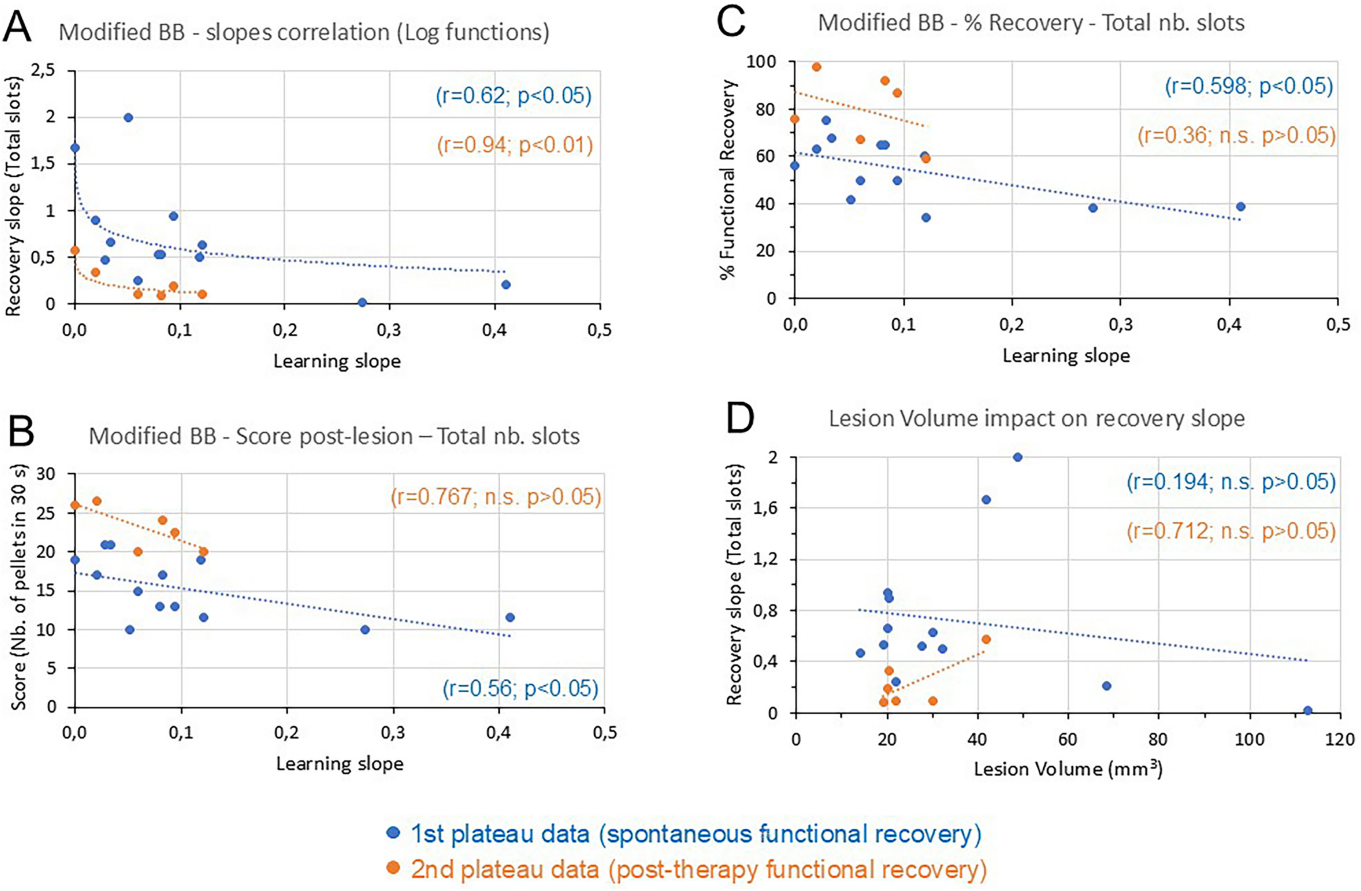
Modified Brinkman Board (BB) data (part 1). **(A)** The *recovery curve slope* was plotted as a function of the corresponding initial *learning curve slope* in the same monkey, separately for the 13 “unique/first plateau” data points (blue dots) and the six “second plateau” data points (brown dots). The manual dexterity scores used to derive the *learning curve slope* and the *recovery curve slope* were those established for the total number of slots retrieved in 30 s (see section “2 Materials and methods”). The data points were fitted with a logarithmic function (dashed blue and brown curves), with the corresponding coefficient of correlations (r = ) and the statistical significance or not of the *p*-values (n.s. is for *p* > 0.05). See Supplementary Table 1 for the precise *p*-values. **(B)** The recovered post-lesion score (total number of slots) was plotted as a function of the initial *learning curve slope*, fitted with a regression line. Otherwise, same conventions as in panel **(A)**. **(C)** The percentage of functional recovery (total number of slots) was plotted as a function of the initial *learning curve slope*, fitted with a regression line. Otherwise, same conventions as in panel **(A)**. **(D)** The *recovery curve slope* was plotted as a function of the M1 lesion volume, fitted with a regression line. Otherwise, same conventions as in panel **(A)**.

Table 1 lists the data relevant for the hypotheses to be tested in the present report (Figure 1). Most of these data (columns 2–12 from the left in Table 1) were published previously (Chatagny et al., 2013; Gindrat et al., 2025; Gindrat, 2015; Hoogewoud et al., 2013; Kaeser et al., 2011, 2014; Liu and Rouiller, 1999; Rouiller et al., 1998; Rouiller, 2026; Roux et al., 2025; Savidan et al., 2017; Schmidlin et al., 2011; Wyss et al., 2013). The new data provided here consist of the “*recovery curve slopes*” (rightmost column in Table 1). In order to test the hypothesis 1 (see Figure 1D), the *recovery curve slope* was plotted as a function of the corresponding motor *learning curve slope* (Figure 2A), separately for the first plateau data (blue dots) and the second plateau data (brown dots). In contrast to the hypothesis 1, the data show for both plateaus that, in the same monkey, steep *recovery curve slopes* were rather correlated with gentle *learning slopes*, and then *recovery curve slopes* tended to decrease as a function of increasing *learning curve slopes* (Figure 2A). This overall tendency was best fitted with a logarithmic function, exhibiting a statistically significant coefficient of inverse correlation (*p* < 0.05 for the first plateau data and *p* < 0.01 for the second plateau data; see Supplementary Table 1 for precise *p*-values).

To test the hypothesis 2 (Figure 1), the post-lesion motor performances were confronted to the motor *learning curve slopes*, considering the median total score post-lesion (Figure 2B) or the percentage of functional recovery for the total scores (Figure 2C). In contrast to the hypothesis 2, the total scores post-lesion tended also to be inversely correlated to the motor *learning curve slopes* (Figure 2B); this was true for both the first plateau data (blue dots) and the second plateau data (brown dots). The two subpopulations data were fitted with a regression line, exhibiting a statistically significant coefficient of correlation (*p* < 0.05) for the first plateau data, but not for the second plateau data (Figure 2B and Supplementary Table 1). In contrast to clinical studies in which only the post-lesion behavioral data are available, the advantage of the present monkey model is to have access to the pre-lesion behavioral data. As a result, the functional recovery of manual dexterity can be expressed by a percentage value, by dividing the post-lesion score by the pre-lesion score. As shown in Figure 2C, the percentage of functional recovery for the total score tended also to be inversely correlated to the motor *learning curve slopes* (coefficient of correlation statistically significant only for the first plateau data: see Supplementary Table 1). Nevertheless, the Figures 2B, C indicate that both parameters reflecting the post-lesion performance yielded consistent data, contradicting the hypothesis 2. Although comparable tendencies to the total scores (Figures 2B, C) were observed when plotting as a function of *learning curve slopes* the post-lesion score or percentage of functional recovery for the horizontal scores or the vertical scores separately (Table 1), the corresponding coefficients of correlation were not statistically significant (not shown).

Finally, one may ask whether the volume of the M1 lesion impacts on the *recovery curve slope*, with a tentative expectation that steep *recovery curve slope* and best recovered motor performance should be present when the M1 lesion was modest in size. The data are presented in Figure 2D, where the *recovery curve slopes* were plotted as a function of the M1 lesion volumes, again separately for the first plateau data and the second plateau data. For the first plateau data (blue dots), the above prediction was not verified, with a poor correlation between these two parameters. The second plateau data, in contrast, exhibited a tendency rather opposite the above prediction, though not statistically significant (*p* > 0.05; see Supplementary Table 1).

**FIGURE 3:**
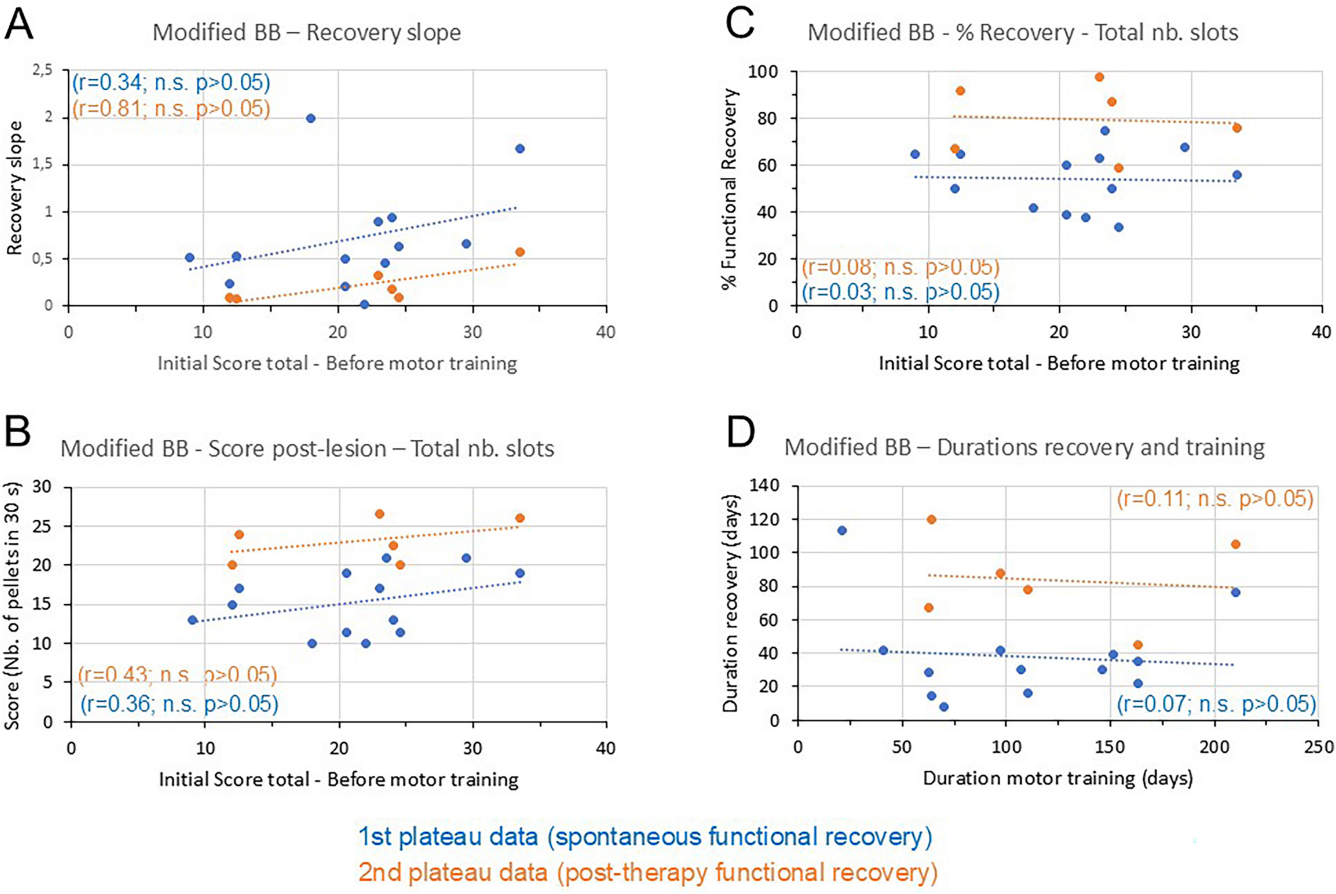
Modified Brinkman Board (BB) data (part 2). **(A)** The *recovery curve slope* was plotted as a function of the corresponding *early initial score* (see Figure 1A) in the same monkey, separately for the 13 “unique/first plateau” data points (blue dots) and the six “second plateau” data points (brown dots). The manual dexterity scores used to derive the *recovery curve slope* were those established for the total number of slots retrieved in 30 s (see section “2 Materials and methods”). The data points were fitted with a regression line (dashed blue and brown curves), with the corresponding coefficient of correlations (r = ) and the statistical significance or not of the *p*-values (n.s. is for *p* > 0.05). See Supplementary Table 1 for the precise *p*-values. **(B)** The recovered post-lesion score (total number of slots) was plotted as a function of the *early initial score*, fitted with a regression line. Otherwise, same conventions as in panel **(A)**. **(C)** The percentage of functional recovery (total number of slots) was plotted as a function of the *early initial score*, fitted with a regression line. Otherwise, same conventions as in panel **(A)**. **(D)** The *duration of functional recovery* (number of days) was plotted as a function of the *duration of motor training* (number of days), fitted with a regression line. Otherwise, same conventions as in panel **(A)**.

The data presented above focused on the here newly introduced parameter of *recovery curve slope* after M1 lesion and its relation with the initial motor *learning curve slope* when the monkey was intact and exposed for the first time to the modified Brinkman Board task (Figure 2A). The motor *learning curve slope* was quite variable among the monkeys (Table 1), indicating significant interindividual differences with respect to margin of progression during the motor learning process. However, in the context of a supplementary analysis, one may also consider the early intrinsic motor capacity of each monkey before the initial motor learning phase, which was given by an “early initial score” (Table 1 and Figure 1A), interpolated as previously reported (Kaeser et al., 2014). Is this early initial score, established for the total number of slots, correlated to some extent to the functional recovery properties after M1 lesion? This supplementary question, not directly related to the main hypotheses of the study, is addressed in Figure 3. The *recovery curve slope* tended indeed to be correlated to the early initial score before motor training, with a tendency of increasing *recovery curve slopes* associated to increasing early initial scores (Figure 3A), close to be statistically significant for the second plateau data (*p* = 0.05072), but not for the unique/first plateau data (Supplementary Table 1). A weak correlation was also observed when plotting the post-lesion median total score as a function of the early initial score (Figure 3B), but this was only a trend (not statistically significant for both plateaus). There was no relationship between the percentage of functional recovery after M1 lesion and the early initial score (Figure 3C). Similarly, there was no correlation between the duration of functional recovery and the duration of the initial motor learning phase (Figure 3D).

## Discussion

Numerous reports derived from studies conducted in non-human primates described the effect of a lesion of the motor cortex, as well as the functional recovery properties with possible mechanisms involved (Barbay et al., 2015; Borgognon and Rouiller, 2023; Bottenfield et al., 2021; Cole and Glees, 1954; Dancause and Nudo, 2011; Dancause et al., 2005, 2006; Darling et al., 2009, 2010, 2013; 2014; 2024; Eisner-Janowicz et al., 2008; Friel et al., 2005, 2007; Frost et al., 2003, 2022; Gindrat, 2015; Glees and Cole, 1950; Hamadjida et al., 2012; Hoogewoud et al., 2013; Kaeser et al., 2011; Le Friec et al., 2021; Liu and Rouiller, 1999; McCann et al., 2025; McNeal et al., 2010; Medalla et al., 2020; Moore et al., 2010, 2012, 2013; 2016; 2019; Moreau-Debord et al., 2021; Morecraft et al., 2016; Murata et al., 2008, 2015; Nudo and Milliken, 1996; Nudo et al., 1996; Nudo, 2013; Orczykowski et al., 2018, 2019; Passingham et al., 1983; Plautz et al., 2003, 2016, 2023; Rouiller et al., 1998; Rouiller, 2024, 2026; Savidan et al., 2017; Wyss et al., 2013; Xerri et al., 1998; Yamamoto et al., 2019). In brief, the perilesional territory as well as intact non-primary motor areas (premotor cortex mostly) play a role in the functional recovery and, in addition, a re-arrangement of the connectivity from and to several motor cortical areas was observed [see (Gindrat et al., 2025; Rouiller, 2024) for a review]. As expected, the studies mentioned above focused on the degree of functional recovery post-lesion, as expressed by the score reached at the plateau of functional recovery. Depending on the M1 lesion size, on the types of behavioral readouts, as well as on the absence or presence of various interventions (e.g., rehabilitative training, treatments, etc.) the functional recovery of manual dexterity was sometimes complete (100% back to pre-lesion score), while it remained incomplete in other cases. Similarly, the reported durations of functional recovery until reaching the post-lesion plateau were quite variable among those studies, ranging from a couple of weeks to about 3 months. Among the 13 monkeys included in the present report, the duration of spontaneous functional recovery ranged from 8 days to about 4 months, to reach either the unique plateau or the first plateau of functional recovery (Gindrat et al., 2025; Gindrat, 2015; Kaeser et al., 2011; Roux et al., 2025; Savidan et al., 2017; Wyss et al., 2013). This large duration variability is, at least in part, due to variability in M1 lesion size and in percentages of largely incomplete functional recovery of manual dexterity. The durations of functional recovery for the second plateau (days from the lesion itself) are not meaningful, as they depend on the precise time points at which the treatments were applied, especially in the 4 ANCE treated monkeys (Kaeser et al., 2011; Roux et al., 2025).

In contrast to the previous reports mentioned in the preceding paragraph, the clear originality of the present study is to provide quantitative data related to the functional recovery time course, in the form of a *recovery curve slope* (as illustrated in Figure 1; data listed in Table 1). For the unique plateau or first plateau of recovery, it corresponds to the slope of the line connecting the first day of score deviation from zero until reaching the plateau, as defined quantitatively earlier (Kaeser et al., 2011). For the second plateau, the slope of the line connecting the score deviation from the first plateau to the onset of the second plateau (Figure 1C). An even more rare originality of the present report is the attempt to correlate for each monkey the *recovery curve slope* to the initial motor *learning curve slope*, when the monkeys in the pre-lesion intact state were exposed for the first time to the same behavioral task (modified Brinkman Board task). Such pre-lesion motor *learning curve slopes* data were not reported earlier in the large palette of studies listed in the bibliography, except in our previous report, from which they were derived (Kaeser et al., 2014). For the first time, Figure 2 thus shows an inverse correlation between the initial motor *learning curve slope* and the *recovery curve slope* after unilateral M1 lesion, for the same manual dexterity task and for the same monkey, in contrast to the hypothesis tested (Figure 1D). This observed inverse correlation, as opposed to an anticipated direct correlation, suggests that the functional recovery time course is most likely not a kind of repetition of the motor learning time course, as depicted by the hypothesis 1 in Figure 1. The data of Figure 2 show that, both for the first plateau and for the second plateau of functional recovery, the *recovery curve slopes* did not increase as a function of increasing motor *learning curve slopes* (as depicted in Figure 1D to illustrate hypothesis 1). On the contrary, there was rather a tendency for an inverse correlation, best fitted with a logarithmic function (Figure 2A). The hypothesis 1 of Figure 1 was thus not verified. Furthermore, there was also an inverse correlation between post-lesion scores and motor *learning curve slopes*, in contradiction to the hypothesis 2 depicted in Figure 1. Finally, the M1 lesion volume was shown to strongly impact on the degree of functional recovery of manual dexterity (Gindrat et al., 2025; Gindrat, 2015; Kaeser et al., 2011; Roux et al., 2025; Savidan et al., 2017; Wyss et al., 2013). In contrast, the M1 lesion volume did not affect the *recovery curve slope*, at least for the unique or first plateau of recovery (Figure 2D).

In a recent report about functional recovery after M1 lesion in macaques (Bottenfield et al., 2021), it was shown that female monkeys recovered more quickly than males. The sex of the 13 monkeys included in the present study is listed individually in Table 1. The *recovery curve slopes* calculated here (Table 1) did not show such a trend toward steeper *recovery curve slopes* in females, as the ranges for each sex largely overlapped. Age was also shown in macaques to affect the motor function of the hand (Moore et al., 2010), with a decrease of performance in monkeys older than 15 years old. The age of the monkeys included in the present study is indicated in Table 1, with a majority of them (*n* = 8) ranging between 3.5 and 5 years old at the time of the M1 lesion. The other five monkeys were older, but all less than 15 years old, suggesting that age presumably did not strongly impact on the present data.

It is important to emphasize here that the inverse correlations observed in Figures 2A–C in no case means causation. Indeed, it would not be justified to conclude that steep initial learning would directly cause slow recovery from M1 lesion, essentially because the present study is impacted by the strong limitation of a low number of subjects. In spite of the attempt to increase the number of spontaneous recovery data points by considering the first plateau in the six “treated” monkeys (see Table 1; Rouiller, 2026), the results presented in Figures 2, 3 are restricted to subgroups of 13 and six data points (first and second plateaus, respectively). Future investigations are needed in order to expand the number of subjects in order to confirm the inverse correlation between the slopes of initial motor learning curve and of motor recovery curve after M1 lesion. Along the same line, the second plateau data points reflecting the effect of a treatment (*n* = 6) were further stratified between the two distinct treatments (two anti-Nogo-A antibody treated monkeys and four ANCE treated monkeys), although the two treatments share several properties of functional recovery enhancement, justifying their pooling (Rouiller, 2026). Moreover, the conclusions derived from Figure 2 cannot at that step be generalized to motor control at large, as it applies to the specific field of manual dexterity, if not to the specific modified Brinkman Board task. Whether the inverse correlation between the slopes of initial motor learning curve and of recovery curve after M1 lesion would also be verified in other manual dexterity tasks (Badi et al., 2021; Barra et al., 2022; Bottenfield et al., 2021; Cole and Glees, 1954; Cole, 1952; Eisner-Janowicz et al., 2008; Gindrat et al., 2025; Kaeser et al., 2013; Klüver, 1935; Lawrence and Kuypers, 1968; Le Friec et al., 2021; Murata et al., 2008; Nudo et al., 1992, 1997; Pizzimenti et al., 2007; Plautz et al., 2003; Savidan et al., 2017; Schmidlin et al., 2011) remains an open question. Another possible confounding factor is the precise age of the monkeys at which they were first confronted to the modified Brinkman Board task, to determine the motor *learning curve slope*. Ideally, an age around 3–5 years old would be optimal, corresponding to juvenile adults. This condition was met in nine out of the 13 monkeys included in the present study (Kaeser et al., 2014); four monkeys were older (6–7 years old) when they were initially trained to the task. It is unclear how this age disparity may have affected the present results. Similarly, there was disparity in the time interval between the end of the initial behavioral training and the M1 lesion: it ranged from 0.5 to 2.5 years in nine monkeys, while in the other four monkeys the time interval was 4, 4.5, 5.5, and 8 years, respectively (years rounded to 0.5 year). Finally, as the experiments were conducted on distinct cohorts of 2–4 monkeys over nearly two decades, there were inevitable slight variations in the housing conditions of the monkeys (to comply with newly introduced ethical regulations), as well as in term of preliminary habituation to the behavioral set-up (Kaeser et al., 2014), again without feasible estimation about possible impact of these variations on the results. To demonstrate causation from the inverse correlations displayed in Figures 2A–C, it would require the application of advanced statistical approaches, such as regression analyses, which may support causal inferences, although a larger number of monkeys is needed to apply such statistics, as mentioned above. Furthermore, additional control experiments would have probably been required to demonstrate causation between initial learning and functional recovery from M1 lesion. Such control experiments were not planned in the original design of the study 2–3 decades ago.

The present report emphasizes how precious is the model of non-human primates in order to investigate the mechanisms of functional recovery of manual dexterity after motor cortex lesion, as well as the possibility to test various therapeutic approaches to enhance the functional recovery. Indeed, non-human primates exhibit a great proximity to humans with respect to the organization of the motor cortical areas (Borra et al., 2010; Bufacchi et al., 2023; Dum and Strick, 2002; He et al., 1993, 1995; Luppino and Rizzolatti, 2000; Strick et al., 2021), to the motor descending pathways (Lawrence and Kuypers, 1967; Lemon, 2008, 2016; Rathelot and Strick, 2009) and to the exquisite capacity to use independently hand fingers underlying manual dexterity (Courtine et al., 2007; Lawrence and Hopkins, 1976; Lawrence and Kuypers, 1968; Lemon, 2019). In the present report, it was exceptionally possible to investigate a putative correlation between motor learning time course (in the intact state) and post-M1 lesion recovery time course. Such a comparison is most likely impossible in human studies, as clinical investigations on stroke patients do not have access to the motor learning data for the same patients, before the pathological event and for the same motor tasks used later in the post-lesion rehabilitative training.

The inverse correlation between the slopes of pre-lesion motor learning curve and post-lesion recovery curve may suggest that a steep initial learning corresponds to an optimization of motor performance, which may limit subsequent motor flexibility in case of M1 lesion later in life. At that step, such interpretation remains speculative, and not supported by the learning data reported earlier (Kaeser et al., 2014). There was no correlation between the slope of the initial learning curve and the score at pre-lesion plateau (Kaeser et al., 2014). Indeed, some monkeys exhibited a steep initial learning, but only during a short time period, resulting in an intermediate score at pre-lesion plateau. In contrast, monkeys with less steep initial learning curve, but during a longer learning period may reach higher score at pre-lesion plateau. Moreover, there was a rather large variability across monkeys with respect to their “naïve” score preceding the initial motor learning phase (3*^rd^* column from right in Table 1). In other words, steep initial learning curve did not necessarily mean high pre-lesion performance in terms of score and, as a consequence, optimal pre-lesion motor performance (Kaeser et al., 2014). During initial learning, it is likely that the corticospinal/corticomotoneuronal system, originating from M1, plays a major role. As a result of M1 lesion, the corticospinal projections from intact premotor areas (PM, SMA) may be engaged, but they are known to be less efficient than that from M1 (Maier et al., 2002; Schmidlin et al., 2008), although post-lesion compensatory sprouting may enhance the influence of the intact PM and/or SMA (McNeal et al., 2010). Previous tract-tracing experiments from M1 lesion monkeys (Borgognon and Rouiller, 2023; Fregosi et al., 2018; Gindrat et al., 2025; Rouiller, 2024) have shown that the cortical projections from PM onto subcortical motor centers (corticorubral and corticoreticular) are strongly downregulated, as compared to intact monkey. As a result, after M1 lesion, the subcortical motor centers may function more independently from the cerebral cortex, exerting via their reticulospinal and rubrospinal projections a control on hand motoneurons post-lesion quite different from the pre-lesion situation. This post-lesional particularity may contribute to the inverse correlation reported here, resulting from different mechanisms involved either in initial learning (cortical control dominance) or in functional recovery from M1 lesion (enhanced subcortical control).

## Conclusion and future directions

On the contrary to what may have been expected, the time course of functional recovery of manual dexterity after M1 lesion in the adult does not appear to resume the time course of an initial motor learning for the specific modified Brinkman Board task. In other words, the present study rather supports the notion that “steep initial motor learning” for a manual dexterity task in the intact state is not necessarily correlated with a more rapid and better functional recovery after M1 lesion; on the contrary, “gentle initial motor learning” is rather correlated with steep motor recovery slope following M1 lesion, as far as manual dexterity is concerned. Indeed, the present data show that “gentle initial motor learning” for a manual dexterity task tended to exhibit steeper functional recovery curves after M1 lesion, as well as a higher recovered score. This conclusion suggests that mechanisms involved in initial motor learning in the intact state are most likely different from motor re-learning mechanisms underlying functional recovery following motor cortex lesion. In the context of rehabilitative training, this conclusion suggests that the most relevant motor tests and strategies may not necessarily be those identified in intact subjects learning new motor tasks. However, the principles governing motor learning are numerous and complex (Maier et al., 2019) and how they interact among each other in the context of motor re-learning along a rehabilitative procedure in patients remain to be investigated further.

## Data Availability

The original contributions presented in this study are included in this article/Supplementary material, further inquiries can be directed to the corresponding author.

## Supplementary material

Supplementary Video 1 Typical daily behavioral session of the modified Brinkman Board task performed by a macaque monkey (macaca fascicularis). Using one hand (the right one here), the monkey has to retrieve food pellets from a board containing 50 slots (25 oriented vertically and 25 horizontally). A “total” score was derived, corresponding to the total number of pellets successively retrieved during the first 30 seconds of the test, in the present case 25. The total score of 25 consists of 12 vertical slots and 13 horizontal slots successively retrieved during the first 30 seconds. Pellets are retrieved by performing the precision grip, by opposing the thumb and the index finger.
