# Supplementary material for: Is there a correlation between functional recovery of manual dexterity after motor cortex lesion and initial motor learning slope in the intact state?
Source: Front Syst Neurosci. 2026 Mar 17;20:1754760. doi: 10.3389/fnsys.2026.1754760 (PMC13036146; doi:10.3389/fnsys.2026.1754760)
Supplement: Supplementary file 1 [file Table_1.docx]

**Supplementary Table 1**

Precise p values for the correlation plots shown in Figures 2 and 3, derived from the Pearson calculator (www.socscistatistics.com).

|  | P values for first plateau | P values for second plateau |
| --- | --- | --- |
|  |  |  |
| Figure 2A | **0.02379 *** | **0.00529 **** |
| Figure 2B | **0.04656 *** | 0.07511 |
| Figure 2C | **0.03087 *** | 0.48333 |
| Figure 2D | 0.52538 | 0.11247 |
| Figure 3A | 0.25569 | 0.05072 |
| Figure 3B | 0.22695 | 0.39475 |
| Figure 3C | 0.9225 | 0.88026 |
| Figure 3D | 0.82024 | 0.83567 |

First plateau corresponds to data points in blue in Figures 2 and 3.

Second plateau corresponds to data points in brown in Figures 2 and 3.

In bold, statistically significant p values at level p<0.01 (**) or p<0.05 (*).
